# Living the everyday of dementia friendliness: Navigating care in public spaces

**DOI:** 10.1111/1467-9566.13442

**Published:** 2022-02-06

**Authors:** Katie Brittain, Cathrine Degnen

**Affiliations:** ^1^ Population Health Sciences Institute Newcastle University Newcastle UK; ^2^ School of Geography, Politics and Sociology Newcastle University Newcastle UK

**Keywords:** carers, dementia, environment, materialities of care, public places, relational places, technologies, UK

## Abstract

Dementia friendly communities are a priority for international policymaking aimed at tackling the social exclusion of people living with dementia. However, what constitutes a dementia friendly community is not well defined nor understood. In this article, we explore what constitutes the enactment of care in a dementia friendly community, focusing on commercial, leisure public places. Through qualitative interviews with carers in the North East of England, we examine how elements of social and material environments shape meaningful everyday practices of care outside the home. Drawing from the literature on materialities of care, we examine three everyday activities: eating out, going to the cinema and shopping. Maintaining such activities in public is part of keeping on with normal family life, but they can also expose individuals to stigmatising judgements by outsiders. Despite this, a complex array of material things, people, places and immaterial qualities such as ambience can come together to make care possible. We suggest there is a need to promote a less rigid, more flexible ethos in these public places. Through a recognition of the relational materialities of care, public spaces could do more to become places where people living with dementia can continue to feel connected and included.

## INTRODUCTION

The attempt to create ‘dementia friendly communities’, an ethos that exhorts community and social settings to ‘share responsibility for ensuring that people living with dementia feel understood, valued and able to contribute to their community’ (Alzheimer's Society, 2021), is rapidly growing in international policy agendas (Department of Health, 2015; World Health Organization, 2017, 2018). Strategies to create dementia friendly communities are intended to tackle the discrimination and social exclusion that people living with dementia^1^ often experience in their everyday lives. To counter this, dementia friendly policy documents encourage all community members—local government, local businesses, service providers and the public—to take responsibility for ensuring the inclusivity of people living with dementia. Whilst a laudable vision, what actually constitutes a dementia friendly community is not well defined and is difficult to determine (Mitchell, 2012). Furthermore, social stigma and negative stereotypes associated with the experience of dementia continue to be wide ranging and pernicious (O’Connor et al., 2018; Scholl & Sabat, 2008). With the push for dementia friendly communities comes the question of how public spaces can become accommodating and meet the needs of people living with dementia and their carers. We seek here to unpack some of these dilemmas by looking more critically at what makes up the practices and aspects of dementia care, and how it is enacted in public shared spaces.

## BACKGROUND

Few studies have examined the everyday experiences of people living with dementia and their carers outside of the home with ‘very little consideration…given to social experiences tied to the neighbourhood of people with dementia or to the relationship of social networks to place and space’ (Keady et al., 2012, p. 150). Detailed, finely grained insights into how people living with dementia engage with their neighbourhoods are noticeably lacking (Ward et al., 2018). The sites where care and support for people living with dementia occur, and the nature of those locations, are critically important; they need also to be able to shift with and be responsive to the changing nature of dementia itself (Davis et al., 2009). We know that people living with dementia can find outside, public spaces both enabling and disabling (Brittain et al., 2010), so a better understanding of what services, facilities, environmental conditions and forms of support are required to ensure the care and comfort of people living with dementia and carers in public spaces is needed. Other authors have recently argued that dementia friendly communities need to be reconceptualised to allow for ‘networked and relational’ aspects to be acknowledged rather than viewing them entirely as ‘fixed, physical or material entities’ (Clark et al., 2020, p. 1). We propose that recent insights developed in the literature on materialities of care are powerful tools for addressing these points, and we draw from that literature to aid us in critically questioning how public, outside places can accommodate the needs of people living with dementia and their carers. In this article, we explore the perspectives of carers who reflect on their experiences of navigating public spaces and everyday activities. The knowledge they have accrued generates invaluable insights that help us better understand how the policy vision for dementia friendly communities translates into actual lived experience.

A materialities of care approach focuses on neglected but crucial aspects of meaningful care, namely the ‘significance of mundane materials as part of social practice’ (Buse et al., 2018, p. 244). Uniting both new materialist theories in STS and work on material culture in health‐care settings (Buse et al., 2018), this body of work foregrounds the deeply relational character of care, one that emerges through a complex array of people, things and places (Cleeve, 2020). This perspective calls attention to the significant role of objects in practising care, making visible the otherwise overlooked ‘“ordinary,” tacit and non‐verbal aspects of care practices’ and highlights how ‘materialities are not merely a backdrop for care interactions, but play an active role in constituting relations of care’ (Buse et al., 2018, p. 245). Materialities are thus core to the relational practices of care for ‘it is clear that materialities are not just what care passes through but rather what makes relationships, and therefore the potential for care, possible’ (Brownlie & Spandler, 2018, p. 267). As such, taking a materialities perspective permits a closer, critical interrogation of the contested concept of ‘care’ itself (Buse et al., 2018), helping us ‘reimagine what is important for occasions to actually be caring’ (Latimer, 2018a, p. 380). It permits us to conceptually untether care from grand narratives of ‘the more heroic aspects of health care’ (Latimer, 2018a, p. 385) and instead concentrate on what supposedly banal practices and mundane objects achieve. Lastly, exploring the role of materialities allows an examination of intersections between the immaterial and material, thus opening up the possibility that more intangible qualities, including the atmospheric and ambient, contribute to care practices (Buse et al., 2018).

A wide range of sites and kinds of care are considered within the materialities of care literature. A subset of this work looks specifically at dementia. Researchers in this field have explored the deep significance of material objects such as handbags and their contents (Buse & Twigg, 2014) and material markings (Cleeve, 2020) for supporting identity and asserting spatial boundaries for people living with dementia; they have demonstrated how materialities can be enrolled in the negotiation of dementia care between carer and cared for (Driessen, 2018); and they have demonstrated the complexity of the array of social and material elements assembled to ensure care (Ceci et al., 2019). This valuable body of work tends to be based within institutional frameworks of health‐care settings with their structured and controlled environments (for instance Buse & Twigg, 2018; Cleeve, 2020; Driessen, 2018) or the domestic home space, which is relatively protected from the potentially judgmental scrutiny of strangers (for instance Araujo et al., 2020; Ceci et al., 2019). We extend and develop these insights by considering dementia care *outside* such settings and look instead at care as it occurs *in public*, shared, communal leisure and commercial spaces. We argue that accessing public places remains a significant element of keeping everyday activities going for our research participants, helping them maintain a sense of ‘normal’ family life. Family outings to commercial and leisure places are highly valued for their crucial importance in sustaining these opportunities for family interactions (Araujo et al., 2020; Ceci et al., 2019). However, the nature and quality of interactions in public settings are also much more unpredictable than within the fixed points of home or residential care. Navigating the public domain means coming into contact with both strangers and people who may not have a sympathetic understanding of dementia nor of how it can alter behaviour and shape social interactions. As we explore below, using shared public spaces makes visible the performative and relational nature of many activities conducted in leisure and commercial settings which, if not carried out ‘correctly’, can become judged by outsiders.

The materialities of care literature, in tandem with the policy vision of dementia friendly communities, pushes us to consider how the relationship of people, things and places enmeshed in care is enacted outward and beyond the relatively controllable spaces of home or institutional care and into more unpredictable public settings. Indeed, attention in the broader dementia care literature is often focused on indoor private spaces and how these support a person's independence and autonomy, whilst ‘the public outdoor world is rarely conceived of as a dementia setting’ (Blackman et al., 2003, p. 361). We know from our previous research that people living with dementia can be disadvantaged because of the physical and social environments in which they live, due to a predominant focus around risk management and the clinical emphasis on biomedical ideas of dementia (Brittain et al., 2010). We also know that the outdoors has a notable significance in the lives of people living with dementia, particularly when their confidence, or lack of, in accessing outdoor spaces restricts them from doing so (Duggan et al., 2008), and that familiarity, accessibility, comfort and safety all influence whether an outside environment is dementia friendly (Duggan et al., 2008; Mitchell et al., 2003). But what remains unclear is the question of how care itself is enacted in a dementia friendly community.

To address this, we explore care as it is practised in everyday places during three ordinary activities in public places: eating out, going to the cinema and shopping. We seek from the perspective of carers to critically explore what is at stake in their efforts to ensure these activities continue for the people they live with and care for. We focus in particular on examples of both sustained processes and practices of successful care, as well as moments where it fragments or feels under threat. We are inspired by Latimer's thinking on materialities of care as a ‘complex interaction between bodies, persons and things, of affect and relationality’ (Latimer, 2018a, p. 388), and seek to extend this to dementia care in order to open up the discussion of how dementia friendly communities can support a move away from functional care to an approach that recognises the complex nexus of care Latimer identifies. We additionally seek to build on valuable insights from work by Brownlie and Spandler (2018) in a study that does not focus on dementia but takes a materialities perspective to outside spaces with neighbours and acquaintances. Their research draws our attention to ‘the possibility for small acts of care to emerge in and through sharing the most ordinary of spaces and things’, highlighting how the informal and incidental contribute to practices of care (2018, p. 266). They focus on mundane help from neighbours and acquaintances in everyday life, where ‘low‐level’ care occurs ‘fleetingly’ (Brownlie & Spandler, 2018, p. 257) and ‘around the edges’ (Anderson et al., 2015, cited in Brownlie & Spandler, 2018). This strikes us as particularly important in helping us understand the possibilities for how care might be enacted in public places seeking to build ‘dementia friendliness’. Thus, in exploring what constitutes a dementia friendly community, we suggest here that we need also to consider how informal, mundane, low‐level and fleeting interactions between strangers, objects and social processes that occur in public places shape these experiences and shape the possibilities of care in these places.

## METHODS

The data presented were gathered as part of a larger EPSRC funded study, MyPLACE: Mobility and Place for the Age‐friendly City Environment, that sought to advance the concept of the age‐friendly city through the development of new digital tools, perspectives, evidence, and citizen‐led design. In preparing this article, we draw on a qualitative sub‐study within MyPLACE that explored what places are dementia friendly from the perspective of carers of people living with dementia in a large city in the North East of England. Ethical approval for this study was obtained from Newcastle University Ethics Committee. A combination of convenience and snowball sampling was used to recruit carers to explore their experiences in accessing public spaces and what they found to be dementia friendly. Twenty‐one carers of people living with dementia were recruited through two of the project partners: a dementia friendly cinema initiative and a dementia friendly community alliance. Participants took part in either a face‐to‐face qualitative interview or a focus group conducted within their own homes, a cinema or café, according to their own preference. In offering either focus groups or interviews, the researchers followed the lead of the partner organisations who explained that some participants would feel more comfortable engaging in a group discussion whilst others would prefer one‐to‐one interviews. Both focus group discussions and individual interviews opened with the question ‘what places do you find dementia friendly?’. Responses to this initial opening were explored and developed around themes of how participants carried out everyday activities in public places when caring for a person living with dementia.

All conversations were digitally audio‐recorded with the respondents’ informed consent, and these were then transcribed verbatim, checked for accuracy, and anonymised. The data were analysed using qualitative thematic analysis (Braun & Clarke, 2006) whereby we individually read and re‐read the transcripts, identifying themes as they emerged across the range of participants’ responses. We came together to discuss and compare our interpretations, continuing to develop the analysis. We then returned to the data to explore in greater depth together the themes we had identified. In the data collection, we sought to elicit carers’ perspectives on their experiences of ordinary activities in public places, and their reflections on what made a place dementia friendly or not. In conversation with our participants, three particular activities emerged as important everyday family outings: shopping, eating out, and trips to the cinema. The first two were not activities we specifically asked about, but we had asked about trips to the cinema. This was because one of our partner organisations were developing a dementia friendly cinema programme and sought insights into what they could change to make going to the cinema more comfortable. What stood out for us from our initial analysis was the detail and depth in which all our participants talked about carrying on everyday activities in these public spaces. What became clear to us as we further analysed and discussed the data were the points of similarity and overlap in all three of these activities in public places, specifically in regard to how they were approached, organised and sustained, something we develop in our findings and discussion below.

## FINDINGS: EVERYDAY MATERIALITIES OF DEMENTIA CARE IN PUBLIC PLACES

Our findings highlight the relational, material and immaterial aspects of care where people, things and places come together in ways that can both facilitate and frustrate meaningful everyday practices of care in public places for people living with dementia and their carers. In this section, we work through the detail of our participants’ accounts of each of the three activities in turn. We begin with the example of eating out in public places.

### Eating out

Eating is a powerful example of a core daily activity that can become more challenging in public places for people living with dementia. This is because some symptoms of dementia, like changes in one's ability to recognise everyday commonplace objects, can in turn disrupt accepted norms. Mark for instance speaks about the challenges around eating when his wife, Ruth, started to lose the ability to recognise and use a knife and fork. He now has to ‘put them into her hand and then her memory will do the automatic’. Although changes in eating practices can be managed and accommodated within the home, they become more apparent and visible when eating in public places where eating out is not just a practicality but also an important social activity, and learning as a carer how to ‘work with’ objects according to new parameters presented by the development of dementia can help facilitate this.

For instance, Sheila describes how they choose restaurants based on the menu, as Paul, her husband, needs meals that can be easily eaten by fork. Some foods in contrast break up too readily and Paul struggles to ‘navigate it’, such as fish and chips. Paul hates it when anyone cuts up his food, so as a couple they try to avoid this and are careful with where they eat in public:

There is a limit to what Paul can eat as well. That’s becoming an issue […] To cut things up now is becoming a bit of a challenge. And anything like a fish, it can break up, can’t it? And then he’s really struggling. All my recipes are around using a fork. So that takes such a lot of adjusting though. Because we will all, as a family, we’ll sit down in a restaurant and go, ‘Oh shit, we never looked at the menu’. We struggle to get used to that.

Here Sheila talks about adapting recipes at home so that the meals she prepares are edible for her husband, such as casseroles that Paul can easily navigate. But the passage also highlights how whilst changing needs are seemingly effortlessly accommodated within the home, the effort of managing these needs becomes thrown into stark relief when venturing into public spaces. This is an example where a simple task, eating out, also ‘support(s) other more complex activities in family and social relations’ and how objects and ‘materialities of all sorts’ are essential in holding these social practices together (Araujo et al., 2020, p. 2). For Paul and Sheila, the fork and careful consideration of the menu are the ordinary objects and practices that enable them to eat out in public places as a couple and family. Furthermore, the fork, itself a seemingly trivial material object, becomes the thing through which meals are arranged and planned; for this couple, the fork plays a crucial role in enacting care and in holding the social together.

Similarly, Kathy describes the challenges she and her husband John faced when eating and drinking in cafes and how staff working within these environments need to be understanding and aware of the needs of people living with dementia:

Going out for a coffee and a cake was the thing that we did. … …but some places were a lot better than others. First of all, it really helps if there’s somebody in the staff who’s got some basic understanding that dementia isn’t just about forgetting things.

Kathy further reflects on eating out in public spaces with John who has lost the ability to recognise everyday objects, such as a knife and fork:

Not identifying it, not seeing the position of it. […] the salient thing for him would be an edge. So he would put his cup on the edge of the table. I was constantly asking them [staff] to return funny cups and plates. And if you go somewhere for something to eat and they present you with a big wooden board, “Oh no, please can I have a white plate?” “Oh I don’t think we’ve got any white plates”[…] I took a plate to eat to tolerant places. And that’s another thing, being tolerant of non‐standard eating practices because we struggled for a while with him being able to eat with eating irons. But in the end I just had to give up, it was too much for, you know, it was too much for him, he couldn’t do it. He put his knife, fork, spoon down on the plate and then couldn’t recognise it for what it was. It might as well not have been there. So he was, without realising it, he was eating effectively with his hands, quite well with his hands, and not everywhere is tolerant of that.

In order to ensure that they were still able to go out for coffee and cake together, ‘the thing that we did’, and an important part of their everyday life together as a couple, Kathy highlights how she learned to bring a simple white plate with her as a way of adapting the public space to mirror what worked at home. For this couple, an ordinary, mundane material object, a recognisable plate, holds steady the social practice of eating out in public places together (Araujo et al., 2020). This is not an example of health care in the ‘heroic’ mode as Latimer describes above (2018a, p. 385), but rather of care emerging from the relational intertwining of objects, people and sociality.

A second important point Kathy makes is that some public places are more accommodating of non‐normative eating practices:

There was a time when I was at a café which is not here, where the waitress came over and she came with another knife and fork set, plonked it down in front of him. And I thought, ‘Actually I’m not going to say anything’, and I just let her wander away. I mean, sometimes I have the energy to say ‘Excuse me, do you realise my husband’s got dementia. He’s not wearing a hat with it on but he isn’t just being a slob’.[…] So I mean he would eat anything with his hands, some people are very put off with that […] So being mindful of other customers, I used to put him so he was facing the wall. Which is a shame really, but facing me and I was facing outwards, so that it wasn’t putting off people too much. But there are some places that are, they’re really good. They can see what’s going on and they’re really, really good about it.

Kathy identifies the best alignment of everyday objects (kind of plates) and setting (seating arrangement; sympathetic waitress) in order to manage not just her husband's comfort and their ability as a couple to keep on eating out in public places, but also to manage potential discomfort of observing strangers who ‘don't want to see that kind of thing’. Indeed, well versed in the widespread discrimination and stigma associated with dementia in Western societies (Milne, 2010), Kathy is hyper vigilant about how her husband's behaviour might be ‘read’ negatively by others. Her words point to the ways in which eating in public is a performative act, scrutinised and judged by others present; if not performed correctly, eating risks stigmatising the eater via the scrutiny of judgemental others in the public space. Kathy in response literally, as well as figuratively, puts herself in between strangers and her husband. She does so in order to shield him and her from their view, using her own body to protect their space and facilitate their ability to eat out without fear of being watched and judged. Whilst some observers unreflexively assume he is ‘just being a slob’, others ‘can see what's going on’ and are supportive and accommodating.

It is evident through Kathy's words that part of her caring practices, which ensures they can keep going into public places, is to monitor, assess and log the relative dementia friendliness of these sites, as well as to anticipate and thus intercept in advance the stigmatising of John by outsiders. Once we scratch beneath the surface, what becomes profoundly evident is the remarkable amount of otherwise hidden care work to make possible these everyday activities, as well as the significance of the actions of others (servers, other customers) for shaping the experience. Here we see too how this hidden care builds on and relies on a complex array of material objects, sociality, physical bodies, carer knowledge and carer experience accrued over a long period of time.

### Going to the cinema

Trips to the cinema are the second social activity through which we explore the nexus of people, things, places and everyday practices of care in public places for people living with dementia. Going to the cinema, like going to a restaurant, is often seen as a treat, a way to be with family and friends. However, the atmospheric qualities that shape cinema spaces can become threatening for people living with dementia, with its loud music and darkened cavernous ceilings. Sheila describes the challenges her son, Tom, was experiencing in continuing a shared lifelong love of watching films with his father, Paul. He had continued taking his father to the cinema for many years, until the low light levels started to become a problem:

And then only probably earlier this year [Tom] said “I’m struggling with dad at the cinema because it’s too loud, it’s too dark and we don’t get there because we’re always running late.” … And the lights, you know. So he was trying to get there earlier so that the lights were on a bit. But it was really Paul starting to say “I don’t want to come, I don’t want to go.” And we knew why.

In Sheila, Paul and Tom's case, the cinema has for many years been a bit of family ‘glue’, something that has been an important bond and site of connection. It remains a valued shared family outing that requires thought and care to carry out, but is worth it to ensure that it still happens.

But atmospheric characteristics of the cinema environment (reduced lighting, immersive sound, requirement to arrive at a precise time and stay quietly seated) began to prove incompatible with Paul's dementia and threatened to fragment this important social bond and site of care between father and son. Indeed, similar atmospheric qualities were cited as a key challenge by a number of carers to taking a person with dementia to watch films in public. Beth for instance felt that the noise level was ‘ear‐splittingly loud’ and that she wanted to say something to the staff but felt unable to leave her dad alone for a short period of time, whilst Karen mentions if the lights are too harsh and it is too loud then it can become a scary environment for someone with dementia. In contrast, she says:

The dementia friendly cinema is brilliant because it's too harsh taking her to a normal cinema; too harsh, noisy, loud. It's scary.

Other atmospheric considerations and challenges that shape the experience of film‐going for our research participants include the distracting qualities of the advertisements played at the beginning of the film flagged up by Olivia, a formal carer:

I think a lot of people at certain stages find it hard to concentrate […] “I can’t follow this storyline.” They might think that is the beginning of the film. I think cutting [the adverts] out so it is just one continuous thing so they can settle into it quickly.

Thus ambient, atmospheric qualities of public places emerge as a key concern through these reflections on cinema‐going and how care is enacted. Of particular note are how adjustments to sound are made so that it is not too ‘harsh, noisy’ or ‘ear‐splitting’, or to avoid light levels being ‘too dark’, or to consider what might be distracting, such as adverts before a film can shape these experiences. These aspects bring to into focus what Buse et al identify as ‘the intersections between the immaterial and material’ (2018, p. 245), seen here in action via the example of the cinema and its ‘more intangible qualities of materialities such as atmosphere and ambiance’ (Buse et al., 2018, p. 245). Recognising the relational aspects of these immaterial qualities—and how they can be shaped—can support successful care for people living with dementia and their carers; discounting them in turn frustrates and blocks care.

In addition to atmospherics, a number of carers talked about how the person living with dementia wanted to sing or dance in the cinema and how this is often not viewed favourably by outsiders. The parallels with our material above about eating out are evident, as non‐normative behaviour risks being judged and stigmatised by outsiders. Alison mentions how she had to try and get her auntie to stop singing because another cinema goer had shouted at her:

Actually, my auntie who had dementia quite a long time ago, I took her to the theatre, we went to see “My Fair Lady” and she was sat singing along to one of the songs. A lady behind her shouted at her and she said she was ruining her whole theatre experience. I had to try and get [my aunt] to be quiet.

In contrast to this unpleasant encounter, Fran highlights how going to watch a film, whether it is in a cinema or in one that is provided within a hospital, can be calming or therapeutic for a person with dementia. She recounts how her mum enjoyed the activity, singing, and was also able to nap. Fran is reluctant to take her to other cinemas because she does not want her mum to be viewed ‘in a reduced way’, as not being equal to others. In so saying, she echoes the concern of Kathy in the previous section on eating out, and like Kathy, Fran is working hard to ‘shield’ her mum from the stigmatising gaze and potential for judgemental assumptions of strangers in public spaces.

Given how significant trips out to the cinema can be for some of our participants in maintaining continuity and familial social ties, attending to atmospheric qualities as well as extending the parameters of “permissible” behaviour can help enable what care might look like in a dementia friendly setting. But given the public nature of cinemas, as with eating out, the intangible relational qualities at play here also include unknown others. Carers anticipate a stigmatising gaze from these outsiders, a perception of stigma for not ‘controlling’ or ‘eliminating’ dementia in this anti‐ageing contemporary culture (Latimer, 2018b) and they work hard to insulate their loved ones from it. This in turn highlights how the agentive role Buse et al. (2018) identify within the relational intersection of the social, material and immaterial are in evidence here—the cinema not simply the setting or ‘backdrop’ of care, but indeed, actively fashioning the relations of care itself.

### Shopping

In our third and final example, we focus on shopping. Returning to Kathy, we learn from her reflections on sustaining her husband's independence and sense of usefulness. These had been put in jeopardy because he was becoming lost, disorientated, and would forget the purpose of his shopping trips:

At one stage, when we moved down here, John still went out on his own. […]. Anyway, one regular thing that he used to do, because he felt useless, I mean in the early days he said he wanted to commit suicide and that upset me a lot […] he used to go out most days and get the bread from Tesco’s […] And what I did was, because […] he’d come back and go ‘I forgot, I think I was going to the shop and I’ve forgotten’, bread […] What I did was, I gave him a big, colourful, shopping bag, which is not too feminine so he didn’t feel too bad. […] I took a photograph of the kind of wholemeal bloomer that we always got and I printed it life‐size and I laminated it and I put it in the shopping bag. And I had a word with one of the guys at Tesco’s and I said ‘my husband comes in to buy bread’, I’ll give him £1, I know it’s 90p so he can’t buy the £1.50 special one, and it’s one coin, and he’s got the picture in his bag. I was worried he would get locked up for shoplifting, you know.

Kathy describes a number of steps she took to help John continue to access everyday public places like Tesco's, a large supermarket chain. This includes selecting a bag that is easily identifiable to him as ‘his’; taking a reference photograph of their normal wholemeal bloomer and making it sturdy through lamination; and speaking with the staff and raise their awareness of John's dementia in order to facilitate his ability to shop successfully. She also draws on her lived experience and accrued knowledge of John to support this, such as a shopping bag chosen because it is ‘not too feminine’ which he might not like, and organising coins because 90p will invisibly guide him away from buying the ‘£1.50 special one’.

Evident here in this one small example is how much effort and thought Kathy has put into making it possible for John to continue to navigate everyday shared public spaces, and to protect his sense of usefulness when he had previously felt suicidal. Reminiscent of how Ceci et al. (2019) describe the steps family take to manage care at home of people living with dementia and ‘work out highly specific ways to live their lives—a practice of tiny, multiple adjustments, localness and multiple paths’ via a ‘gathering of people and things’ (2019, p. 1215), Kathy has adjusted and reflected on what material objects, social interactions and practices support her husband to continue shopping for their favoured loaf of bread. Via materialities of care, she knits together mundane items (shopping bag, laminated cards, coins) and other people (‘one of the guys at Tesco’ that she's had a word with; and the imagined character who might accuse him of shoplifting). Kathy does this to enable John's self‐worth—key for working against his suicidal thoughts—but she also assembles care via seemingly inconsequential objects, via her lived knowledge and via enrolling other people because she is worried John will otherwise be arrested for shoplifting. In so doing, she takes steps to mitigate against that threat of strangers who she imagines will assume the worse of him and who will not understand that he has dementia. Anticipatory work and management strategies are thus part of enacting care here, but Kathy also treads a complex line, juggling between the socially stigmatising aspects of John's condition as well as enabling him to continue everyday activities in public places.

In a second example, the physical environment of shopping malls emerges as a key consideration for Ruth and Mark who we started the article with. Over time, Ruth has started to be uncomfortable using escalators and more recently, she has started to refuse to get into lifts, but Mark, her husband and carer, cannot use the stairs because he becomes breathless. This convergence has proved tricky:

She won’t go down escalators which is understandable. She won’t even go up them now which is a problem. Even when we tried going up in the lift she normally doesn’t bother but she began to quaver over doing that. I thought “how the hell are we going to get up and downstairs in [name of large shopping centre] if we can’t use the stairs or the lift?”

But the relative social awareness of some retailers in comparison with others is also an important consideration. Mark explains about how he finds certain retailers more helpful and accommodating of his wife's needs:

I must say in the shops, getting back to your theme (DFC), Marks and Spencer's and Waitrose are the ones we frequent mostly. They are unbelievably helpful. I always go back, “(I say I’m) trying to get a hat” there for example for her. I say “Look, don’t think it’s strange me buying her clothes but she has got Alzheimer’s.” They said “Don’t worry” then take over and help tremendously…they are always helpful in those two shops.

In shopping for a hat together, Mark feels more confident in some shops than others to explain that his wife has dementia, and to solicit the help of staff knowledgeable in women's fashion. When he says ‘unbelievably helpful’ and recalls their words of ‘don't worry’, he helps us see how they have created a comfortable environment for both his and her needs.

Similarly, Rose speaks about a landmark department store in the city centre that ‘has always been there’ in her life, and which is a familiar place to her and her husband. She recounts how she lets staff there know that her husband has dementia so that if he becomes disorientated, they may be able to help:

For years we have always gone to (name of shop) for coffee and breakfast, every Wednesday morning you will find a group of my family. (Name of shop) has always been there in my life… (when I worked as a childminder) I looked after twins. .... The women were always ready to carry your tray or to help you to get highchairs and that. I have gone in there for years and years and years. They know you. They all know you. I did say to them a few months back, I told them about Alan. I said, “If he ever comes in,” because it is the only place he will go now for a coffee. He can get the bus into town and he can go for a coffee and get his newspaper and he can get the bus back home again. He will phone me up and say “What time is the bus?” because he can’t work numbers out. But I have said to them, “Look he does have Alzheimer’s. If he does come in and you think he is a bit, you know, just take care of him and make sure he gets out okay.” Because sometimes he can’t work the buttons on the lift and he won’t go on the escalator because he doesn’t feel safe on it. The ladies in the restaurant know about him. Plus the lady who is always on the newspapers, she knows.

The regular, habitual, banal aspects of shopping in a familiar place over many years are highlighted here. Indeed, it is arguably these same characteristics that mean shopping is an important everyday event and activity to maintain for people living with dementia and their carers. That is to say, it is the actual *doing* of these shopping activities which is linked to a person's sense of wellbeing, such as for John and Alan. But also evident in these examples are the deeply social and relational elements that can make shopping pleasurable and make places feel ‘known’, an expression which surpasses a simple familiarity with place, but instead points to the webs of relation through time that connect people and place (Degnen, 2013). For Rose and Alan, there is an attachment to a specific shop which holds particular deep biographical significance and continuity across their lives. Such ontological continuity is enhanced by the women working in the restaurant and at the newspaper stand who Rose and Alan ‘can count on being noticed and acknowledged as well as given support “round the edges”’ as Brownlie and Spandler (2018, p. 263) write about in their account of informal helping and ‘by the by’ moments that piece together to shape mundane care in the public realm. For Alan, like Ruth, the materiality of lifts and elevators have become difficult and feel unsafe. But in ‘fleeting’ moments, others help ‘just take care of him’. The carers draw our attention to their role in facilitating these everyday public activities, as well as logging the significance of others whose empathy supports them to protect these significant activities in their lives for them and for the person living with dementia. These brief, informal moments of interaction and sociability are significant: ‘the ladies in the restaurant know about him’; ‘one of the guys at Tesco's’; the staff who help Mark ‘tremendously’ and ‘take over’ the tricky business of hat buying—these moments are evidence of how relative strangers too can help support care that is enacted in public places through micro and yet consequential acts of kindness and understanding, in conjunction with material objects and the ambience of ‘known’ places.

## DISCUSSION: THE INTERSECTING ELEMENTS OF CARE

This article has sought to better understand the question of what constitutes the enactment of care in a dementia friendly community. Our thinking here has been influenced by recent work in the literature on materialities of care and what it demonstrates about how nominally inconsequential things come to matter for care practices. We have explored here how the material, social, physical and atmospheric aspects of lived environments can come together relationally to facilitate ‐ or hinder ‐ people living with dementia and their carers in public spaces. In particular, we have explored the ways in which different material objects (such as plates, forkable food, a shopping bag) and the qualities of places (the right ambient qualities, locations that are ‘known’) ‘extend from their apparent inert object‐ness and work to constitute different kinds of practices and relations’ (Araujo et al., 2020, p. 5) which render care in public commercial and leisure spaces successful or not.

A limitation of our study is that we focus solely on the insights of family members who are caring for people living with dementia. This means that we are not able to represent the immediate lived experiences of those individuals with dementia themselves, nor their own perspectives on how their relationships with outside public spaces may have changed over time. There is scope to explore this through other research methods that more directly engage and involve those living with dementia. Whilst recognising this, we also believe it is important not to demote the significance of carers’ accounts of navigating public places. We propose that the richness in the carers’ narrative accounts of everyday activities raise important questions in the debate around what constitutes a dementia friendly community, and argue that their perspectives complement the work of others who have focused on the accounts of people living with dementia themselves.

One of the insights we have gained through our approach has been to make visible the extraordinarily detailed planning and trial‐and‐error approach adopted by carers. They have experimented with objects and practices (forkable food, recognisable plates and ambient conditions) in their domestic care practices, building knowledge that they then use to bridge their way into less controllable environments in public. Through the narrative accounts of our participants, we see too how the strong rules of normative behaviour around eating, shopping and behaviour in the cinema can be tricky to navigate in public because of perceived negative judgments by strangers. And yet, we also see that it is when small aspects of the comfort and ease of the home space can be reproduced when out and about (even in a micro, momentary fashion) that dementia friendliness is facilitated, such as via light levels or familiar objects. We are struck by the extent to which carers find creative and imaginative ways to harness the relational power of materialities and socialities to ensure continued use of public space for themselves and their partners. The path of least resistance might be to retreat to more controllable spaces of the home. However, we argue that these families are actively doing something quite radical by continuing to stake a claim to their entitlement to public space despite a range of social and practical challenges they face.

Managing the perceived scrutiny by others that can occur when outside of the home, carers take on the role of assessing, monitoring and evaluating the interactional parameters of public places. This is a crucial and yet heavy load of hidden labour on the part of carers. It includes ‘shielding’ those they care for from stigmatising judgments by using the materiality of their own bodies and material objects as part of care work. This hidden labour also includes accruing detailed, bespoke knowledge over time about what array of materials, practices and ambience ‘works’ for those they care for—arrays that vary substantially from individual to individual, and demonstrating great scope to better accommodate such heterogeneity in shared settings. Carers invest in working against a rigidity in public spaces, through adapting public space to accommodate non‐normative practices. Some settings make this more possible than others, such as the ‘tolerant’ restaurants that permit a white plate to be brought in from home, menus with ‘forkable’ dishes (a category of food itself only identified and named via Shelia's carefully attuned attention to Paul's changing needs), employees helping a male carer shop for a hat for his wife, a cinema that does not frown upon singing and informal empathetic help ‘by the by’ from staff and strangers.

These elements forge moderately less rigid environments, freed of some of the unspoken ‘rules of behaviour’ which are generally very restrictive and intolerant, and can enable everyday practices such as eating, shopping and watching a film to continue for carers and people living with dementia. That is to say when materialities and socialities are working well together, public spaces appear to become more flexible in a way that is more inclusive and dementia friendly, and in ways that facilitate meaningful care. Cafes, for instance, could conceivably offer a choice of plates as they do a choice of bread type for toast, and more care could be given to varying atmospheric qualities such as light and noise. Additionally, these mundane materialities of care are not always orchestrated nor even highly visible. They instead often occur in ‘fleeting moments’, ones that might be hardly perceptible, but which have significant, tangible impacts nonetheless. Taken together, these examples demonstrate the relational configuration of objects, places, bodies and knowledge being employed to enact care, identifying the elements that enable and restrict a person living with dementia during their everyday activities.

Lastly, some of the challenges presented by policies of dementia friendliness are that they inadvertently promote a blanket vision that masks multiple points of distinction, such as gender differences. John's shopping bag that is chosen because it is ‘not too feminine’ and Mark's anxiety that the staff might think ‘it's strange me buying her clothes’ are prime examples of how gender roles inflect experiences and practices of carers as they seek to navigate public space with their partners. Examining such social forms of distinction across a wide range— including age, sexuality, race, ethnicity, class—were beyond the scope of this study, but they demand further attention and consideration. A further related point is to not lose sight of the commercial nature of these leisure spaces we have been describing. Their responsibility for care is arguably not the same as other leisure sites like libraries which are embedded in a nominal civic of duty of care. A blanket policy vision imagines all settings as equally invested (and able to invest) in community wellness. We suggest a more nuanced discussion is needed around care in public spaces, one that takes into account the different contexts and types of public spaces.

## CONCLUSION

The policy vision of creating dementia friendly communities in order to tackle the discrimination and social exclusion often experienced by people living with dementia is a commendable response. However, more needs to be known about how people living with dementia and their carers experience public spaces, the complexities of how care is practised and what makes up care in public settings. This has pushed us to critically question how dementia care is enacted outward and beyond the fixed points of the private domestic realm or institutional settings. We have followed the lead of our participants in focusing on three everyday activities in public settings, namely eating out, trips to the cinema and shopping. In doing so, we demonstrate how carers of people living with dementia develop a series of creative, bespoke techniques to both maintain their use of public places as well as protect against stigma experienced there, often building on knowledge gained first in the home space to bridge into public settings.

Materialities of care emerge as a critical aspect of managing the potential stigmatising judgements made by strangers when a person living with dementia's behaviour disrupts strong social norms in public places. Our participants’ accounts make visible the ways in which a complex array of material things, people, places and immaterial qualities such as ambience can come together to make care possible in public. They also showcase the creative strategies and accrued knowledge they leverage to contend with the threat of stigma in public, as well as the significant hidden labour of carers in working across the interconnected realms of the social and material.

We argue that important lessons can be learned from imagining dementia friendly communities as a complex intersection where care both makes—and is made by—social and material relations. Through a broader recognition of the relational materialities of care, public spaces could do more to become places where people living with dementia can continue to feel connected and included. This would mean commercial, leisure public places promoting a less rigid, more flexible ethos which is less bound by unspoken rules of behaviour. The relational, materialities of care play a considerable part in whether or not this vision can or will be truly realised.

## AUTHOR CONTRIBUTION

**Katie Rhian Brittain:** Conceptualization (equal); Data curation (equal); Formal analysis (equal); Funding acquisition (equal); Writing – original draft (equal); Writing – review & editing (equal). **Cathrine Degnen:** Conceptualization (equal); Data curation (equal); Formal analysis (equal); Funding acquisition (equal); Writing – original draft (equal); Writing – review & editing (equal).

## ENDNOTE

^1^
‘Dementia’ itself is a contested category and is an all‐encompassing label that includes a number of sub‐types of cognitive impairment (Moreira & Bond, 2008). Whilst we recognise using the term ‘people living with dementia’ might risk masking the intense heterogeneity and variety of lived individual experiences of dementia, we also recognise the value of the phrase for acknowledging the lived experiences of individuals who are experiencing a wide range of symptoms and clinical manifestations of a complex disease. This term attempts to give a voice to those experiences as well as to emphasise the subjectivity and personhood of those individuals.
